# Suicidality Among Healthcare Workers in Lebanon: Associations With Childhood Adversities Amid Recent Overlapping Crises

**DOI:** 10.3389/ijph.2025.1608725

**Published:** 2025-10-13

**Authors:** Josleen Al Barathie, Mary-Lee Wakim, Joe Allabaky, Rayane Osman, Elie Karam

**Affiliations:** ^1^ Institute for Development, Research, Advocacy, and Applied Care (IDRAAC), Beirut, Lebanon; ^2^ Department of Psychiatry and Clinical Psychology, Saint George Hospital University Medical Center, Beirut, Lebanon; ^3^ Department of Psychiatry and Clinical Psychology, Saint George University of Beirut Faculty of Medicine, Beirut, Lebanon

**Keywords:** suicide, childhood adversities, Beirut blast, COVID-19, economic collapse, healthcare workers

## Abstract

**Objectives:**

Healthcare workers (HCWs) face heightened suicide risk due to occupational stressors and other proximal and distal factors. To our knowledge, this study is the first study in Lebanon and among the first globally to examine the association between childhood adversities and suicidality among HCWs within overlapping national crises.

**Methods:**

We conducted a cross-sectional analysis of a cohort study among 390 HCWs in Lebanon. Using an online survey, data included sociodemographics, Beirut port blast, adulthood trauma, economic collapse, COVID-19, network/support, childhood adversities, mental health (PHQ-9/PCL-5), substance use, prior health and suicidality. Analyses in Stata used bivariate and stepwise logistic regressions to determine parsimonious predictors of suicidality in past-two-week and lifetime suicidality.

**Results:**

Childhood emotional neglect and depression emerged significantly predicted suicidality in the past-two-weeks. Younger age, PTSD due to childhood trauma, PTSD related to a loved one’s illness and depression were significantly associated with lifetime suicidality. Contrary to previous findings, COVID-19 stressors and financial difficulties were not retained in the final model.

**Conclusion:**

HCWs suicidality is associated with early-life adversities and trauma. Findings highlight the need for targeted interventions.

## Introduction

“We define *suicide* as the act of intentionally ending one’s own life” [1]. As explained by Nock et al. [1], suicidal behaviors, described as non-fatal, are a group of three specific classifications: suicidal ideations; ideas to deliberately terminate one’s own life, suicidal planning; model of terminating one’s own life and suicidal attempt; actively participating in behaviors aiming to end one’s life without culminating in death.

Suicide remains under-researched in the 22 Arab World countries [2]. Large-scale studies show relatively low suicide rates, with the Eastern Mediterranean region reporting 6.4 per 100,000, below the global average of 9.0 per 100,000 [3]. In Lebanon, the age-standardized suicide rate in 2019 was 2.8 per 100,000.

Lebanon’s suicide data is primarily derived from the Internal Security Forces (ISF), which is the only official source on this issue [4]. Recent ISF data shows a 21.7% increase in suicide-related deaths in 2023 compared to 2022, and a 46% increase compared to 2021. These figures are nearing the levels recorded in 2019, which had the highest suicide rates of the past decade in Lebanon.

The rise in suicide cases may reflect actual increases or improved reporting mechanisms, but cultural stigma and underreporting obscure the true extent. Suicide remains a deep-seated taboo in Lebanon, affecting both the general population and healthcare workers (HCWs).

Suicidality among HCWs is influenced by several key factors [5]. First, stigma surrounding mental health in the healthcare profession often discourages workers from seeking help, as they fear judgment, loss of credibility, or professional repercussions. In particular, characteristics like perfectionism, though often valued in the workplace, may intensify suicidality [6]. Moreover, access to mental health resources is limited due to logistical challenges, including long wait times, improper mental health coverage, and heavy work schedules. Confidentiality concerns also play a significant role. Additionally, the high-stress nature of healthcare work—caring for critically ill patients, handling heavy workloads, delivering difficult news, and coping with death and suffering—contributes to chronic stress and emotional exhaustion [7]. HCWs' access to lethal means, such as medications, increases the risk of fatal suicide attempts [8]. Ethical dilemmas and moral injury also play a critical role in the mental health challenges faced by HCWs [9]. Moral injury refers to distress experienced when actions, or inactions, contradict one’s ethical or moral beliefs. This can arise in situations such as making life-altering decisions about allocating limited resources, like ventilators. While moral injury is not classified as a mental illness, its effects—such as negative self-perception, guilt, and shame—can lead to mental health issues like depression and suicidality.

In recent years, growing global attention has highlighted the mental health challenges faced by HCWs. Research has since focused on identifying risk and protective factors influencing suicidality in this group.

Major factors associated with increased suicidality among HCWs include various demographic, occupational, psychological, and social factors. Younger age [10–17], living alone [10, 18], a history of mental disorders [11, 16, 19], prior suicide attempts [10, 13, 20–24], working excessive hours [25–28] personal and loved ones physical health issues [10, 19, 29–31], poorer subjective health [10, 20, 23, 24, 32], change in vitamin D levels [33], personal and family problems [30, 34], exposure to sexual abuse [35], harassment [35, 36], or domestic violence [35], conflicts at work [30], financial stress [10, 19, 21], low organizational justice [30], and workplace discrimination [37–39], as well as not feeling support from superiors [11] are all associated with increased suicide risk. Poor sleep quality [10, 12, 16, 20, 23, 24, 40], frequent nightmares [10, 20, 23, 24, 32], workplace violence [41], and a perceived lack of control over working conditions — role conflict and degrading work experiences [42] — also contribute to increased odds. Additionally, suicidality was relatively high for participants characterized by several COVID-19-related factors, such as fear of the virus [43], lack of confidence in overcoming COVID-19 [15], COVID-19 infection in the community [15], perceived lack of institutional preparedness [19], reports of unreasonable demands and complaints from patients or their families [44], willingness to attend gatherings [15], need for psychological assistance before the outbreak or during the epidemic remission period [15, 29], not owning enough equipment to manage patients [45, 46], having changed to a specific COVID-19-related work location [11, 46, 47], or having an infected friend or family member [10, 19, 23, 29]. Psychological distress and poor mental health [10, 18, 19, 35, 48], including depression [10, 20, 29, 32, 48–52], anxiety [10, 11, 18, 20, 29, 48–51, 53, 54], post-traumatic stress disorder (PTSD) [10, 55], burnout [10, 12, 13, 45, 56, 57], exhaustion [58], severe general distress [14], and psychotropic drug use [21, 55] further heightens the likelihood of suicidality.

Several protective factors have been identified also. Having strong social support from family, friends, or colleagues [15, 16, 23, 29, 30, 42, 59], a high monthly income [10, 19, 21], confidence in standard precautions [14], and overall life satisfaction [60] are associated with a lower likelihood of suicidality.

Nonetheless, several factors yielded inconsistent results including gender, having children, having been isolated or quarantined due to COVID, having direct contact with people infected with COVID-19, seniority in the job, being married, substance use, smoking, education level, COVID‐19 infection history, history of psychiatric service contact [10, 11, 13–16, 18, 19, 21, 25–27, 29, 31, 32, 34, 35, 40, 45, 47–50, 54, 58, 59, 61–70].

While numerous studies have identified various risk and protective factors for suicidality among HCWs, many of these factors exhibit inconsistencies, and remain underexplored in the global literature. To address these gaps, to the best of our knowledge, this is the first study of its kind in Lebanon and among the first internationally to examine: 1) all types of suicidalities, 2) both clinical and non-clinical healthcare professionals, 3) two-time dimensions of suicidality (past 2 weeks and lifetime), and incorporates data on childhood and life adversities in relation to suicidality among HCWs in our context and worldwide. Additionally, it examines the impact of three major stressors—the COVID-19 pandemic (*placed an unprecedented burden on the healthcare system, straining resources and exposing HCWs to sustained physical and psychological stress*), the financial meltdown (*the Lebanese currency was depreciating drastically, losing 98% of its value by 2022*), and the Beirut Port blast of August 4, 2020 (*resulted in substantial casualties, injuries, and destruction; was categorized as the largest non-nuclear explosion in modern history, resulting in over 200 fatalities, 6,000 injuries, and the displacement of 300,000 individuals* [71]).

## Methods

### Study Design and Participants

The study population included health workers employed at Saint George Hospital University Medical Center (SGHUMC) which is located near the port, was extensively damaged and became a critical site for both victims and responders. The population included clinical, administrative, and supportive roles. All participants were aged 18 years or older [72].

The dataset used in this study is a cross-sectional analysis of the third wave of a large cohort research project where data was collected across multiple waves [73]. The first wave, conducted 9–15 days post-blast, was initiated during a mandatory COVID-19 testing campaign for hospital staff [74]. Data were collected face-to-face using self-administered questionnaires at the testing site. Waves 2, 3, and 4 of data collection occurred at 21–27 days, 6–7 months, and 2–2.5 years post-blast, respectively. These waves utilized an online platform to distribute surveys via email, SMS, WhatsApp, and QR-coded letters [72]. Waves 1 and 2 focused primarily on the Beirut port blast and collected data on acute stress disorder symptoms only. In contrast, wave 3 included additional mental health screening tools and a broader range of variables relevant to the present analysis. Of the 1927 HCWs who participated in wave 1, 808 participated in wave 3 (response rate = 41.8%) [72]. For the present analysis, only participants from wave 3 with complete data on all key variables were included, resulting in a final sample of 390 participants.

### Instruments

#### Sociodemographic

Participants provided sociodemographic details, including age, gender, education, household composition, and profession (clinical vs. non-clinical).

#### Beirut Blast

Exposure to the Beirut Port Blast was assessed using a 9-item inventory capturing: location during the blast, personal injury, difficulty accessing medical care, injury or death of loved ones, damage to home, participation in rescue efforts, and seeing mutilated or dead bodies. Each exposure was assigned a weight (0–100) by an expert panel; the median weight per item was used to calculate a cumulative weighted exposure score. Full methodology was published elsewhere [74].

#### Previous Trauma Exposure and Reaction

Participants reported prior trauma exposure, including major accidents, life-threatening illness (self or loved ones), deaths of loved ones, and exposure to war or armed conflict. Childhood trauma items included physical abuse, emotional neglect, and sexual abuse. All responses were binary (Yes/No). PTSD-like reactions (lasting ≥1 month and causing distress or impairment) were also assessed and coded dichotomously.

#### Economic Situation

Financial strain was assessed with questions on changes in financial stability, lifestyle (basic/leisure), and household contributors over the previous year. Responses were categorized as “No change” and “Yes change.”

#### COVID-19 Exposure Score

A composite COVID-19 exposure score captured weighted pandemic-related experiences and concerns, including: violence due to being a healthcare worker (Yes/No), proximity to COVID-19 patients (Yes/No), adequacy of Personal Protective Equipment (PPE; Yes/No), death of a loved one (Yes/No), isolation (Yes/No), patient deaths, stigma (Yes/No), triage decisions (Yes/No), fear of infection or transmission (Yes/No), trust in institutions (5-point Likert scale: 1 = not at all to 5 = extremely), and training adequacy (Yes/No). Weights were based on expert-derived importance ratings.

#### COVID-19 Workplace

Participants indicated whether they had been reassigned to new teams or duties since the pandemic began (Yes/No).

#### Network and Support

Network and support were assessed with two 4-point Likert-scale items evaluating perceived emotional and practical support from colleagues and loved ones (1 = strongly disagree, 2 = disagree, 3 = agree, 4 = strongly agree). In addition, participants indicated whether they felt a need for psychological support related to the COVID-19 pandemic, financial situation, or the Beirut explosion, with responses categorized as Yes/No.

#### Mental Health Disorders

PTSD symptoms were measured using the validated Arabic version of the PTSD Checklist for DSM-5 (PCL-5), with items rated on a 4-point Likert scale [75, 76]. A score of ≥2 considered symptom endorsement. Diagnosis followed DSM-5 criteria: at least one B item, one C item, two D items, and two E items. In this study, the scale is reliable with high internal consistency (α = 0.92).

Depressive symptoms over the past 2 weeks were assessed with the Arabic Patient Health Questionnaire-9 (PHQ-9) which has been rigorously validated in Lebanon across various population and demonstrated strong psychometric properties, confirming its utility to screen for depression in our context [77, 78]. Items were scored 0–3, yielding a total score (0–27). A cut-off of ≥10 indicated probable depression (sensitivity = 0.85; specificity = 0.89). Internal consistency was good (α = 0.89).

#### Substance Use

Substance use was assessed with items addressing changes in the use of tobacco (cigarettes, chewing tobacco, cigars) and alcoholic beverages (beer, wine, liquor) during the pandemic. Responses were categorized as: no use, decreased use, same use, or increased use since the pandemic.

#### Prior Health

Participants reported any chronic physical conditions (Yes/No) or pre-pandemic mental health diagnoses (Yes/No).

#### Suicide (Outcome)

The study’s primary outcome was suicide-related behaviors, assessed across two-time frames: the past 2 weeks and lifetime. Questions focused on four domains: wishing for death, suicidal thoughts, planning methods, and suicide attempts. To create a composite variable for analysis, a response of “yes” to any of the domains was categorized as a positive indication of suicide-related behavior. This composite variable, termed “any suicidality,” was generated separately for the past 2 weeks and lifetime experiences, enabling an assessment of both acute and historical suicide risk. Validated suicidality instruments were not included in the parent cohort due to survey length and feasibility constraints. Instead, these *ad hoc* items were selected to capture the core domains of suicidality while minimizing participant burden.

### Statistical Analysis

In this study, missing data were addressed using complete case analysis (CCA), whereby only participants with no missing values on any of the variables included in the analysis were retained. CCA was chosen for its straightforward implementation as it does not involve complex imputation methods that could introduce bias if the assumptions of missingness mechanisms are violated [79].

The prevalence of suicide-related behaviors was calculated for both the past 2 weeks and lifetime (ever) experiences. For each domain—wish, thought, plan, and attempt—the frequency (N) and percentage (%) were reported. Additionally, the same measures were reported for the composite variable, “any suicidality,” which combined a positive response to any of the four domains.

Descriptive statistics were used to summarize all variables. For continuous variables, the mean and standard deviation (SD) were reported, while for categorical variables, frequency (N) and percentage (%) were presented.

To examine associations between suicidality (in the past 2 weeks and over the lifetime) and individual, trauma-related, and contextual predictors, bivariate logistic regression analyses were conducted. The predictors included sociodemographic characteristics, Beirut Blast exposure, previous trauma exposure and reactions, economic situation, COVID-19-related factors, network and social support, mental health disorders, substance use, and prior health conditions. For each analysis, unadjusted odds ratios (ORs) and p-values were reported.

Finally, a stepwise logistic regression analyses were conducted for “any suicidality” in the past 2 weeks and lifetime to identify the most parsimonious model given the number of predictors relative to our sample size. We acknowledge, however, that stepwise procedures have limitations, including overreliance on statistical criteria; nonetheless, all predictors in the initial models were selected based on a literature review informed by theory. Variables with a p-value <0.05 in the bivariate analysis were included in the multivariable model. The final models retained only variables that remained significant at this threshold, providing adjusted ORs for the key predictors of suicide-related behaviors.

## Results

### Descriptive Statistics

The study population was predominantly female, well-educated, and engaged in clinical professions, with a mean age of 37 years (SD = 12.32) (Supplementary Table S1).

Exposure to trauma was diverse, reflecting Lebanon’s history of conflict and recent catastrophic events.

The Beirut Blast was a major exposure (mean = 167.15, SD = 130.52), with participants reporting a wide range of severity scores (Supplementary Table S1).

Beyond this event, participants reported varied trauma histories, including major accidents (7.84%, n = 28), life-threatening illnesses (5.04%, n = 18: personal and 15.69%, n = 56: loved one), and the loss of loved ones (33.89%, n = 121) (Supplementary Table S1). A history of war-related exposure was common, with more than two-thirds of participants having lived through armed conflicts or explosions (Supplementary Table S1). Childhood adversity was also reported, with experiences ranging from physical abuse (17.37%, n = 62) and emotional neglect (14.85%, n = 53) to sexual abuse (5.32%, n = 19) (Supplementary Table S1). The psychological burden of these experiences was evident in PTSD symptoms, which varied based on the nature of the trauma. Financial strain was common as well.

Despite these challenges, many participants reported strong social support networks, both in the workplace (79.83%, n = 285) and within their personal lives (93.28%, n = 333) (Supplementary Table S1). Mental health screening revealed notable rates of PTSD (19.89%, n = 71) and depression (12.04%, n = 43) among participants (Supplementary Table S1). Changes in substance use patterns were notable, with increases in tobacco (12.32%, n = 44) and alcohol (8.96%, n = 32) consumption reported post pandemic (Supplementary Table S1) Detailed descriptive statistics are presented in Supplementary Table S1.

### Bivariate Analysis

Statistical results for all analyses below are presented in Supplementary Material S1; Supplementary Tables S2,S3.

Age was significantly associated with suicide-related behaviors, with younger individuals exhibiting higher odds of both past 2 weeks (OR = 0.95, 95% CI = 0.91–0.99, p = 0.019) (Supplementary Table S2) and lifetime (OR = 0.91, 95% CI = 0.87–0.95, p < 0.001) suicide-related behaviors (Supplementary Table S3).

Certain types of trauma exposure were significantly associated with suicide-related behaviors. In the past 2 weeks, individuals who had experienced a major accident (OR = 3.36, 95% CI = 1.15–9.77, p = 0.026) or childhood emotional neglect (OR = 3.00, 95% CI = 1.22–7.36, p = 0.016) exhibited higher odds of suicide-related behaviors (Supplementary Table S2). Childhood physical abuse (OR = 2.42, 95% CI = 0.995–5.90, p = 0.051) approached significance (Supplementary Table S2).

Childhood emotional neglect (OR = 3.16, 95% CI = 1.55–6.46, p = 0.002), childhood physical abuse (OR = 3.19, 95% CI = 1.61–6.33, p = 0.001), and childhood sexual abuse (OR = 4.61, 95% CI = 1.71–12.41, p = 0.003) were significantly associated with an increased likelihood of reporting lifetime suicidality (Supplementary Table S3). PTSD symptoms due to childhood trauma exhibited a particularly strong association in lifetime suicide-related behaviors (OR = 38.75, 95% CI = 8.06–186.36, p < 0.001) (Supplementary Table S3). The death of a loved one not related to the Beirut Blast (OR = 2.06, 95% CI = 1.09–3.87, p = 0.025), major accident (OR = 2.55, 95% CI = 1.02–6.40, p = 0.046) and life-threatening physical illness of a loved one (OR = 2.54, 95% CI = 1.24–5.22, p = 0.011) were also significant, while life-threatening personal physical illness (OR = 2.88, 95% CI = 0.97–8.49 p = 0.056) approached significance (Supplementary Table S3). For PTSD symptoms resulting from traumatic events, only PTSD secondary to a loved one’s life-threatening physical illness (OR = 7.14, 95% CI = 2.88–17.73, p < 0.001) and PTSD secondary to death of a loved one (OR = 2.35, 95% CI = 1.03–5.34, p = 0.042) were significant (Supplementary Table S3).

Workplace reassignment during the pandemic was significantly linked to lifetime suicide-related behaviors (OR = 3.10, 95% CI = 1.59–6.06, p = 0.001) (Supplementary Table S3).

Individuals who needed psychological support due to the pandemic (OR = 2.48, 95% CI = 1.08–5.67, p = 0.032) or financial difficulties (OR = 2.32, 95% CI = 1.01–5.39, p = 0.049) had significantly higher odds of suicide-related behaviors in the past 2 weeks (Supplementary Table S2). For lifetime suicide-related behaviors, needing psychological support due to the pandemic (OR = 2.44, 95% CI = 1.27–4.65, p = 0.007) and the Beirut explosions (OR = 2.70, 95% CI = 1.43–5.10, p = 0.002) were significantly associated with increased odds (Supplementary Table S3).

Mental health showed a differential impact across both timeframes. In the past 2 weeks and over the lifetime, depression was significantly associated with higher odds of suicide-related behaviors (OR = 8.96, 95% CI = 3.76–21.34, p < 0.001; Supplementary Table S2) (OR = 5.07, 95% CI = 2.44–10.54, p < 0.001; Supplementary Table S3), while PTSD was not (OR = 1.30, 95% CI = 0.50–3.38, p = 0.594; Supplementary Table S2) (OR = 0.71, 95% CI = 0.30–1.67, p = 0.438; Supplementary Table S3).

Mental health diagnosis prior to the pandemic was significantly associated with suicide-related behaviors, both in the past 2-week (OR = 2.94, 95% CI = 1.04–8.32, p = 0.042; Supplementary Table S2) and lifetime (OR = 4.21, 95% CI = 1.69–10.46, p = 0.002; Supplementary Table S3). Increased tobacco and alcohol use since the pandemic was significantly associated with lifetime suicide-related behaviors (OR = 4.87, 95% CI = 2.27–10.43, p < 0.001), (OR = 3.52, 95% CI = 1.40–8.83, p = 0.007) (Supplementary Table S3). For further information, see Supplementary Tables S2,S3 in Supplementary Material S1.

### Multivariable Analysis

**FIGURE 1:**
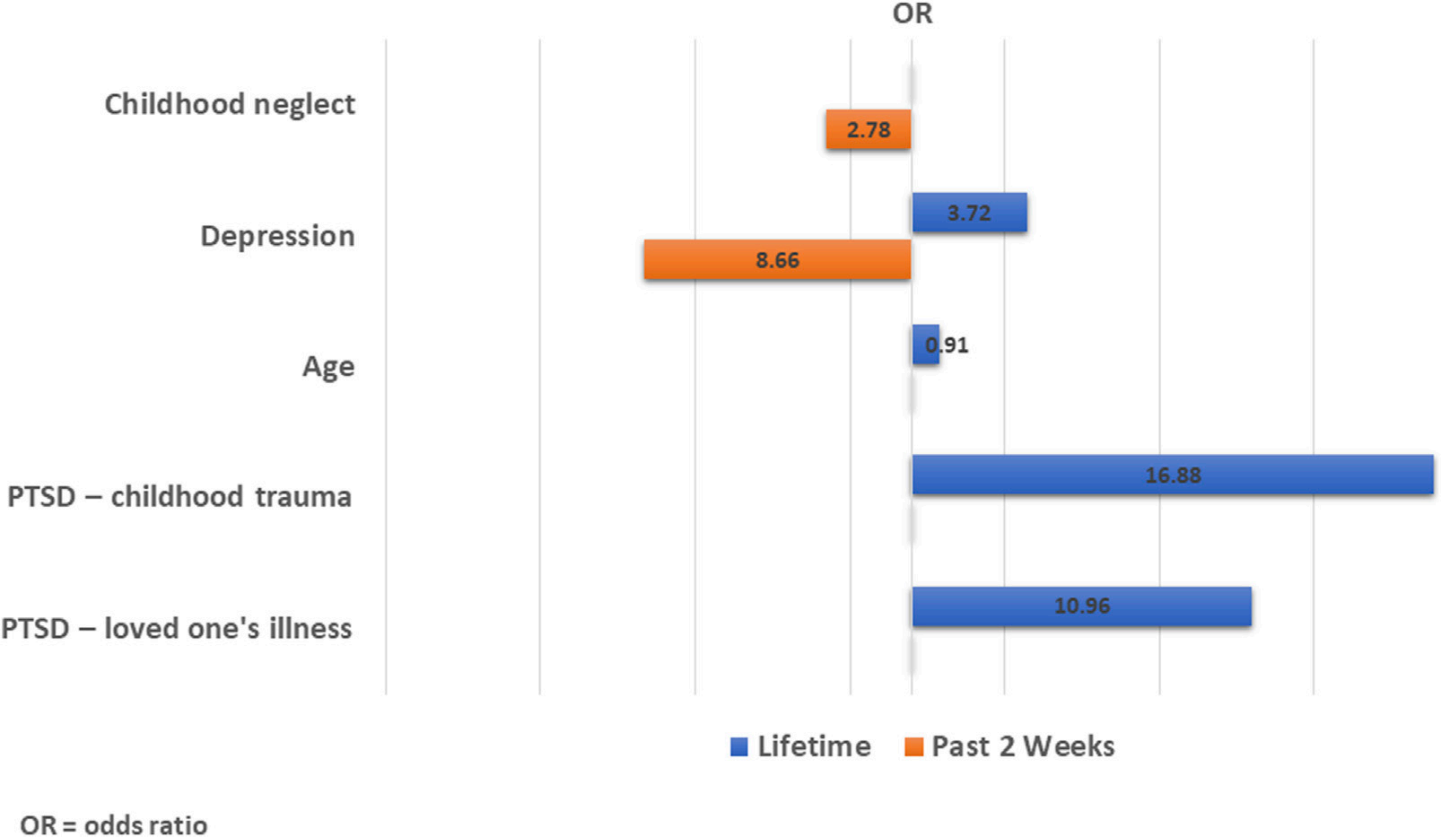
Pyramid of predictors of suicide risk: multivariable stepwise logistic regression (past 2 Weeks vs. lifetime) (Lebanon, 2020-2023).

In the Multivariable analysis using stepwise regression, different factors were retained as significant predictors of suicide-related behaviors for the past 2 weeks (Table 1) and lifetime (Table 2). Childhood emotional neglect (OR = 2.78, 95% CI = 1.06–7.26, p = 0.037) and depression (OR = 8.66, 95% CI = 3.59–20.87, p < 0.001) were significant predictors of suicidality in the past 2 weeks (Table 1). Younger age (OR = 0.91, 95% CI = 0.87–0.96, p < 0.001), PTSD symptoms resulting from childhood adversity (OR = 16.88, 95% CI = 3.16–90.23, p = 0.001) and from a life-threatening physical illness of a loved one (OR = 10.96, 95% CI = 3.27–36.75, p < 0.001) were associated with higher likelihood of reporting lifetime suicidality (Table 2). Additionally, depression remained a strong predictor (OR = 3.72, 95% CI = 1.61–8.58, p = 0.002) of lifetime suicidality as well (Table 2). These findings are also illustrated in Figure 1, which shows the predictors retained as significant in the stepwise models for past 2 weeks and lifetime suicidality.

**TABLE 1:** Stepwise multivariable logistic regression of suicide in the past two weeks (Lebanon, 2020-2023).

Variables	Stepwise logistic regression			
	OR	p-value	95% CI lower	95% CI upper
Childhood neglect				
*No*	Ref			
*Yes*	2.78	0.037*	1.06	7.26
Depression				
*No*	Ref			
*Yes*	8.66	<0.001*	3.59	20.87

OR, odds ratio; CI, confidence interval Ref = reference category.

*p ≤ 0.05.

**TABLE 2:** Stepwise multivariable logistic regression of lifetime suicide (Lebanon, 2020-2023).

Variables	Stepwise logistic regression			
	OR	p-value	95% CI lower	95% CI upper
Age^	0.91	<0.001*	0.87	0.96
PTSD symptoms secondary to childhood trauma				
*No*	Ref			
*Yes*	16.88	0.001*	3.16	90.23
PTSD symptoms secondary to a loved one’s life-threatening physical illness				
*No*	Ref			
*Yes*	10.96	<0.001*	3.27	36.75
Depression				
*No*	Ref			
*Yes*	3.72	0.002*	1.61	8.58

OR, odds ratio; CI, confidence interval; Ref, reference category.

*p ≤ 0.05.

^continuous variable.

## Discussion

The mental health of HCWs has received increasing attention during the COVID-19 pandemic, particularly in relation to suicide risk. However, stressors do not occur in isolation; individuals are embedded in complex contexts shaped by both proximal and distal stressors. Understanding suicide risk among HCWs therefore requires a comprehensive examination of these cumulative and interacting stressors. To the best of our knowledge, this study is the first of its kind in Lebanon and among the first internationally to examine suicide risk among HCWs amidst overlapping collective stressors such as the Beirut port blast, the economic crisis, and the COVID-19 pandemic, while also accounting for childhood and adulthood adversities.

Previous research has predominantly focused on COVID-19-specific stressors, with limited attention to personal and family adulthood adversities [30, 34] and almost none on childhood adversities. Our study addresses this gap by incorporating the extensive literature linking childhood adversity to suicide risk in general populations [80–83]. Research has shown that physical and sexual abuse increases suicidality [84–87], although some studies found no association [88–90], likely due to sample variations, low prevalence or early onset of suicidal behavior [91]. In our study, emotional neglect emerged as a significant childhood adversity predictor alongside PTSD secondary to childhood adversities. This aligns with literature identifying emotional neglect as a powerful risk factor [82, 92, 93], potentially due to its lasting impact on emotional development and self-perception [80].

Although COVID-19-related stressors have been associated with suicide risk in prior studies, they were not retained in our final model. This may suggest that the impact of COVID-19 on suicidality operates indirectly, possibly through their association with depression and PTSD, which emerged as stronger predictors. It may also reflect the greater salience of broader stressors collected through our comprehensive consideration of contextual factors such as the economic crisis and cumulative adversities, which could overshadow the independent association of pandemic-related stressors when considered simultaneously. However, at the bivariate level, several factors such as exposure to COVID-19 cases, patient death, tobacco and alcohol use, stigma and workplace distrust, were significantly associated with suicide risk, aligning with previous studies [15, 19, 62] (Supplementary Material S1, Supplementary Table S4).

Our study also explored subjective financial strain. Rather than relying solely on income, we assessed participants’ perceptions of their financial situation to better reflect individual experience. This is an important departure from conventional approaches, as financial distress is influenced not just by income but also by expectations and obligations. Although subjective financial strain did not remain significant in our final model, this result reinforces the complexity of financial stress and highlights the need for more nuanced assessments in future research.

The meta-analysis spanning 50 years found minimal evidence supporting specific protective factors against suicide, as these factors are rarely examined and generally exhibit weak associations [83]. In our study, protective factors were not retained in the final model. We attribute this to the ad-hoc nature of our assessment, and suggest that 11future research look at various aspects of social networks and support, including tangible, emotional, and affectionate support.

Adulthood adversities such as major accidents, physical illness of a loved one, and the death of a loved one were associated with suicide risk at the bivariate level. At multi-level, only PTSD related to a loved one’s physical illness remained significant. This suggests that although adulthood adversities are impactful, their effects may be more situational and transient compared to the long-term imprint of childhood trauma.

Depression, as expected, was a strong predictor of suicidality, in line with prior evidence [10, 20, 29, 32, 48–52]. Younger age also emerged as an independent predictor of suicide-related behaviors in our sample. This finding is consistent with prior research highlighting increased vulnerability among early-career healthcare workers [10–17], who often face high workloads, limited professional autonomy, and job insecurity. These stressors may heighten susceptibility to psychological distress and suicidality, particularly in the context of Lebanon’s overlapping crises.

A key methodological strength of this study was the use of weighted scores for stressors, enabling a more refined analysis than simple stressor counts. This approach mitigates the oversimplification that arises from merely counting stressors and provides a more nuanced understanding of their impact.

Additionally, our inclusion of multiple layers of adversity (childhood trauma, adulthood events, and collective crises) provided a rich framework for understanding suicide risk in this unique context. Additionally, rather than relying solely on objective indicators of financial hardship, our study emphasized the role of subjective financial strain, highlighting the importance of individual perception in financial stress assessments. Our study also examined individual responses to adversity by including PTSD symptoms, recognizing that the same event may elicit varied psychological impacts. The finding that PTSD related to both childhood adversity and a loved one’s illness were significant predictors highlights the importance of trauma responses over mere exposure.

### Limitations

Despite its strengths, the study has several limitations. Self-reported data may be affected by recall bias or underreporting due to stigma or social desirability—especially for sensitive topics like childhood abuse and suicidal thoughts. Additionally, the cross-sectional nature of the analysis limits causal inference. Important variables such as burnout, marital status, substance use, and anxiety were not included, and PTSD was assessed through a single ad-hoc question, potentially underestimating its complexity. Suicide risk was also measured using ad-hoc items rather than standardized instruments, which may reduce reliability and comparability. Furthermore, our reliance on screening tools rather than clinical diagnoses introduces a risk of misclassification, although such tools are commonly used in population research. Moreover, as the study was monocentric in nature, findings may not be generalizable to all healthcare workers in Lebanon or the broader region. Lastly, sample attrition from 808 to 390 participants raises concerns about selection bias.

### Conclusion

In conclusion, although this study relied on self-reported data, employed a cross-sectional design, did not include burnout, anxiety, marital status, and substance use as predictors, used ad-hoc questions rather than tools to assess suicidality, and was monocentric, it offers valuable insights into the multifaceted stressors that are associated with suicide risk among HCWs in Lebanon during the COVID-19 pandemic and concurrent national crises. Emotional neglect in childhood, depression, younger age and PTSD reactions to childhood and adulthood adversities were key predictors of suicide risk. Our findings emphasize the importance of adopting a life-course perspective when examining suicide risk, incorporating both early-life and recent adversities. Future prevention strategies should integrate trauma-informed care and targeted support for HCWs with a history of early adversity. Researchers should also prioritize validated tools and longitudinal designs to deepen understanding and guide effective interventions.
