# Supplementary material for: Suicidality Among Healthcare Workers in Lebanon: Associations With Childhood Adversities Amid Recent Overlapping Crises
Source: Int J Public Health. 2025 Oct 13;70:1608725. doi: 10.3389/ijph.2025.1608725 (PMC12554631; doi:10.3389/ijph.2025.1608725)
Supplement: Supplementary file 1 [file Supplementaryfile1.docx]

**Journal name:** International Journal of Public Health (IJPH)

**Title**: Suicidality Among Healthcare Workers in Lebanon: Associations with Childhood Adversities Amid Recent Overlapping Crises

**Supplementary Tables S1–S4**

**Table S1: Descriptive statistics**

| **Variables** | **N (%)** |
| --- | --- |
| **Suicidal Behaviors in the past two weeks** |  |
| *Wish* | 23 (6.44%) |
| *Attempt* | 4 (1.12%) |
| *Thought* | 3 (0.84%) |
| *Plan* | 5 (1.40%) |
| *Any Suicide* | 25 (7.00%) |
| **Suicide Behaviors lifetime** |  |
| *Wish* | 42 (11.76%) |
| *Attempt* | 5 (1.40%) |
| *Thought* | 15 (4.20%) |
| *Plan* | 12 (3.36%) |
| *Any Suicide* | 45 (12.61%) |
| **Age^** | 36.76 ± 12.32 |
| **Gender** |  |
| *Woman* | 248 (70) |
| *Man* | 108 (30) |
| **Highest level of education** |  |
| *Incomplete primary schooling* | 6 (1.68) |
| Primary school | 14 (3.92) |
| *Secondary school* | 41 (11.48) |
| *Technical-professional training* | 51 (14.29) |
| *Undergraduate degree (university training)* | 121 (33.89) |
| *Postgraduate studies (master's, doctorate, medical specialty, etc.)* | 124 (34.73) |
| **Living with someone < 18** |  |
| *No* | 217 (60.78) |
| *Yes* | 140 (39.22) |
| **Living with someone >65** |  |
| *No* | 268 (75.07) |
| *Yes* | 89 (24.93) |
| **Living with someone with disabilities** |  |
| *No* | 343 (96.08) |
| *Yes* | 14 (3.92) |
| **Profession** |  |
| *Non-clinical* | 91 (25.49) |
| *Clinical* | 266 (74.51) |
| **Beirut Blast exposure^** | 167.15 ± 130.52 |
| **Major accident (Vehicle, work-related, severe fall etc?)** |  |
| *No* | 329 (92.16) |
| *Yes* | 28 (7.84) |
| **Life-threatening personal physical illness** |  |
| *No* | 339 (94.96) |
| *Yes* | 18 (5.04) |
| **Life-threatening physical illness of a loved one/other** |  |
| *No* | 301 (84.31) |
| *Yes* | 56 (15.69) |
| **Death of loved one(s) not due to Beirut Blast** |  |
| *No* | 236 (66.11) |
| *Yes* | 121 (33.89) |
| **Ever exposed to war/conflict in Lebanon** |  |
| *No* | 116 (32.49) |
| *Yes* | 241 (67.51) |
| **Childhood physical abuse** |  |
| *No* | 295 (82.63) |
| *Yes* | 62 (17.37) |
| **Childhood neglect** |  |
| *No* | 304 (85.15) |
| *Yes* | 53 (14.85) |
| **Childhood sexual abuse** |  |
| *No* | 338 (94.68) |
| *Yes* | 19 (5.32) |
| **Financial Strain** |  |
| *No change* | 111 (31.09) |
| *Yes change* | 246 (68.91) |
| **Basic lifestyle** |  |
| *No change* | 107 (29.97) |
| *Yes change* | 250 (70.03) |
| **Leisure lifestyle** |  |
| *No change* | 68 (19.05) |
| *Yes change* | 289 (80.95) |
| **Number of household contributors** |  |
| *No change* | 227 (63.59) |
| *Yes change* | 130 (36.41) |
| **PTSD symptoms secondary to childhood trauma** |  |
| *No* | 346 (96.92) |
| *Yes* | 11 (3.08) |
| **PTSD symptoms secondary to wars, armed conflicts and explosions** |  |
| *No* | 306 (85.71) |
| *Yes* | 51 (14.29) |
| **PTSD symptoms secondary to a major accident** |  |
| *No* | 352 (98.60) |
| *Yes* | 5 (1.40) |
| **PTSD symptoms secondary to a life-threatening personal physical illness?** |  |
| *No* | 355 (99.44) |
| *Yes* | 2 (0.56) |
| **PTSD symptoms secondary to a loved one's life-threatening physical illness** |  |
| *No* | 335 (93.84) |
| *Yes* | 22 (6.16) |
| **PTSD symptoms secondary to the death of a loved one (excluding the Beirut Port Blast)** |  |
| *No* | 318 (89.08) |
| *Yes* | 39 (10.92) |
| **Job reassignment/new functions since the pandemic** |  |
| *No* | 196 (54.90) |
| *Yes* | 161 (45.10) |
| **Covid-19 Score** | 18.90 ± 7.33 |
| **Supportive colleagues’ network at work** |  |
| *Strongly disagree* | 21 (5.88) |
| *Disagree* | 51 (14.29) |
| *Agree* | 212 (59.38) |
| *Strongly agree* | 73 (20.45) |
| **Supportive loved one’s network** |  |
| *Strongly disagree* | 9 (2.52) |
| *Disagree* | 15 (4.20) |
| *Agree* | 189 (52.94) |
| *Strongly agree* | 144 (40.34) |
| **Need for psychological support due to the pandemic (even if not received)** |  |
| *No* | 266 (74.51) |
| *Yes* | 91 (25.49) |
| **Need for psychological support due to the financial situation (even if not received)** |  |
| *No* | 273 (76.47) |
| *Yes* | 84 (23.53) |
| **Need for psychological support due to the Beirut explosions (even if not received)** |  |
| *No* | 247 (69.19) |
| *Yes* | 110 (30.81) |
| **PTSD** |  |
| *No* | 286 (80.11) |
| *Yes* | 71 (19.89) |
| **Depression** |  |
| *No* | 314 (87.96) |
| *Yes* | 43 (12.04) |
| **Pre-pandemic chronic physical illness** |  |
| *No* | 328 (92.65) |
| *Yes* | 26 (7.35) |
| **Pre-pandemic mental health diagnosis** |  |
| *No* | 340 (95.77) |
| *Yes* | 15 (4.23) |
| **Change in use of tobacco (cigarettes, chewing tobacco, cigars, etc.) since the pandemic** |  |
| *I do not use this substance* | 229 (64.15) |
| *My use has decreased* | 29 (8.12) |
| *My use has remained the same* | 55 (15.41) |
| *My use has increased* | 44 (12.32) |
| **Change in use of alcoholic beverages (beer, wine, liquor, etc.) since the pandemic** |  |
| *I do not use this substance* | 170 (47.62) |
| *My use has decreased* | 50 (14.01) |
| *My use has remained the same* | 105 (29.41) |
| *My use has increased* | 32 (8.96) |

^^continuous variables: mean ± SD^

**Table S2: Bivariable logistic regression of suicide in the past two weeks**

|  | **Simple Logistic Regression** | | | |
| --- | --- | --- | --- | --- |
| **Variables** | **OR** | **p-value** | **95% CI Lower** | **95% CI Upper** |
| **Age^** | 0.95 | 0.019* | 0.91 | 0.99 |
| **Gender** |  |  |  |  |
| *Woman* | **Ref** |  |  |  |
| *Man* | 0.89 | 0.792 | 0.36 | 2.17 |
| **Highest level of education** |  |  |  |  |
| *Postgraduate studies (master's, doctorate, medical specialty, etc.)* | **Ref** |  |  |  |
| *Incomplete primary schooling* | 2.28 | 0.471 | 0.02 | 4.13 |
| Primary school | 0.88 | 0.904 | 0.02 | 7.40 |
| *Secondary school* | 0.29 | 0.238 | 0.01 | 2.33 |
| *Technical-professional training* | 0.23 | 0.164 | 0.01 | 1.85 |
| *Undergraduate degree (university training)* | 1.14 | 0.774 | 0.05 | 4.67 |
| **Living with someone < 18** |  |  |  |  |
| *No* | **Ref** |  |  |  |
| *Yes* | 1.04 | 0.934 | 0.45 | 2.38 |
| **Living with someone >65** |  |  |  |  |
| *No* | **Ref** |  |  |  |
| *Yes* | 0.55 | 0.291 | 0.18 | 1.66 |
| **Living with someone with disabilities** |  |  |  |  |
| *No* | **Ref** |  |  |  |
| *Yes* | 1.02 | 0.983 | 0.13 | 8.15 |
| **Profession** |  |  |  |  |
| *Non-clinical* | **Ref** |  |  |  |
| *Clinical* | 1.40 | 0.515 | 0.51 | 3.84 |
| **Beirut blast exposure^** | 0.9989 | 0.535 | 0.996 | 1.000 |
| **Major accident (Vehicle, work-related, severe fall etc?)** |  |  |  |  |
| *No* | **Ref** |  |  |  |
| *Yes* | 3.36 | 0.026* | 1.15 | 9.77 |
| **Life-threatening personal physical illness** |  |  |  |  |
| *No* | **Ref** |  |  |  |
| *Yes* | 2.88 | 0.114 | 0.78 | 10.71 |
| **Life-threatening physical illness of a loved one/other** |  |  |  |  |
| *No* | **Ref** |  |  |  |
| *Yes* | 1.78 | 0.241 | 0.68 | 4.68 |
| **Death of loved one(s) not due to Beirut Blast** |  |  |  |  |
| *No* | **Ref** |  |  |  |
| *Yes* | 1.33 | 0.505 | 0.58 | 3.05 |
| **Ever exposed to war/conflict in Lebanon** |  |  |  |  |
| *No* | **Ref** |  |  |  |
| *Yes* | 0.49 | 0.091 | 0.22 | 1.12 |
| **Childhood physical abuse** |  |  |  |  |
| *No* | **Ref** |  |  |  |
| *Yes* | 2.42 | 0.051 | 0.995 | 5.90 |
| **Childhood neglect** |  |  |  |  |
| *No* | **Ref** |  |  |  |
| *Yes* | 3.00 | 0.016* | 1.22 | 7.36 |
| **Childhood sexual abuse** |  |  |  |  |
| *No* | **Ref** |  |  |  |
| *Yes* | 1.61 | 0.540 | 0.35 | 7.40 |
| **Financial Strain** |  |  |  |  |
| *No change* | **Ref** |  |  |  |
| *Yes change* | 0.96 | 0.919 | 0.40 | 2.29 |
| **Basic lifestyle** |  |  |  |  |
| *No change* | **Ref** |  |  |  |
| *Yes change* | 1.38 | 0.501 | 0.54 | 3.57 |
| **Leisure lifestyle** |  |  |  |  |
| *No change* | **Ref** |  |  |  |
| *Yes change* | 1.25 | 0.688 | 0.42 | 3.78 |
| **Number of household contributors** |  |  |  |  |
| *No change* | **Ref** |  |  |  |
| *Yes change* | 1.18 | 0.700 | 0.51 | 2.70 |
| **PTSD symptoms secondary to childhood trauma** |  |  |  |  |
| *No* | **Ref** |  |  |  |
| *Yes* | 3.12 | 0.161 | 0.64 | 15.30 |
| **PTSD symptoms secondary to wars, armed conflicts and explosions** |  |  |  |  |
| *No* | **Ref** |  |  |  |
| *Yes* | 1.55 | 0.401 | 0.56 | 4.35 |
| **PTSD symptoms secondary to a major accident** |  |  |  |  |
| *No* | **Ref** |  |  |  |
| *Yes* | all individuals who have PTSD symptoms secondary to a **major accident** reported suicide in the past two weeks |  |  |  |
| **PTSD symptoms secondary to a life-threatening personal physical illness?** |  |  |  |  |
| *No* | **Ref** |  |  |  |
| *Yes* | 13.79 | 0.066 | 0.84 | 227.39 |
| **PTSD symptoms secondary to a loved one's life-threatening physical illness** |  |  |  |  |
| *No* | **Ref** |  |  |  |
| *Yes* | 1.36 | 0.693 | 0.30 | 6.17 |
| **PTSD symptoms secondary to the death of a loved one (excluding the Beirut Port Blast)** |  |  |  |  |
| *No* | **Ref** |  |  |  |
| *Yes* | 1.62 | 0.403 | 0.52 | 4.98 |
| **Job reassignment/new functions since the pandemic** |  |  |  |  |
| *No* | **Ref** |  |  |  |
| *Yes* | 1.91 | 0.126 | 0.83 | 4.38 |
| **Covid-19 Score** | 0.97 | 0.372 | 0.92 | 1.03 |
| **Supportive colleagues’ network at work** |  |  |  |  |
| *Strongly disagree* | **Ref** |  |  |  |
| *Disagree* | 2.32 | 0.307 | 0.46 | 11.62 |
| *Agree* | 0.52 | 0.417 | 0.11 | 2.52 |
| *Strongly agree* | 0.27 | 0.202 | 0.04 | 2.03 |
| **Supportive loved one’s network** |  |  |  |  |
| *Strongly disagree* | **Ref** |  |  |  |
| *Disagree* | 0.25 | 0.290 | 0.02 | 3.25 |
| *Agree* | 0.30 | 0.157 | 0.06 | 1.58 |
| *Strongly agree* | 0.18 | 0.053 | 0.03 | 1.02 |
| **Need for psychological support due to the pandemic (even if not received)** |  |  |  |  |
| *No* | **Ref** |  |  |  |
| *Yes* | 2.48 | 0.032* | 1.08 | 5.67 |
| **Need for psychological support due to the financial situation (even if not received)** |  |  |  |  |
| *No* | **Ref** |  |  |  |
| *Yes* | 2.32 | 0.049* | 1.01 | 5.39 |
| **Need for psychological support due to the Beirut explosions (even if not received)** |  |  |  |  |
| *No* | **Ref** |  |  |  |
| *Yes* | 1.85 | 0.144 | 0.81 | 4.22 |
| **PTSD** |  |  |  |  |
| *No* | **Ref** |  |  |  |
| *Yes* | 1.30 | 0.594 | 0.50 | 3.38 |
| **Depression** |  |  |  |  |
| *No* | **Ref** |  |  |  |
| *Yes* | 8.96 | <0.001* | 3.76 | 21.34 |
| **Pre-pandemic chronic physical illness** |  |  |  |  |
| *No* | **Ref** |  |  |  |
| *Yes* | 2.27 | 0.081 | 0.90 | 5.68 |
| **Pre-pandemic mental health diagnosis** |  |  |  |  |
| *No* | **Ref** |  |  |  |
| *Yes* | 2.94 | 0.042* | 1.04 | 8.32 |
| **Change in use of tobacco (cigarettes, chewing tobacco, cigars, etc.) since the pandemic** |  |  |  |  |
| *I do not use this substance* | **Ref** |  |  |  |
| *My use has decreased* | 3.46 | 0.029* | 1.14 | 10.55 |
| *My use has remained the same* | 0.63 | 0.547 | 0.14 | 2.86 |
| *My use has increased* | 2.13 | 0.172 | 0.72 | 6.31 |
| **Change in use of alcoholic beverages (beer, wine, liquor, etc.) since the pandemic** |  |  |  |  |
| *I do not use this substance* | **Ref** |  |  |  |
| *My use has decreased* | 0.29 | 0.248 | 0.04 | 2.34 |
| *My use has remained the same* | 1.19 | 0.716 | 0.46 | 3.07 |
| *My use has increased* | 2.68 | 0.089 | 0.86 | 8.31 |

*^OR = odds ratio
CI = confidence interval^*

*^Ref = reference category^*

*^*p ≤ 0.05^*

*^^continuous variable^*

**Table S3: Bivariable logistic regression of lifetime suicide**

|  | **Simple Logistic Regression** | | | |
| --- | --- | --- | --- | --- |
| **Variables** | **OR** | **p-value** | **95% CI Lower** | **95% CI Upper** |
| **Age^** | 0.91 | <0.001* | 0.87 | 0.95 |
| **Gender** |  |  |  |  |
| *Woman* | **Ref** |  |  |  |
| *Man* | 0.84 | 0.637 | 0.42 | 1.71 |
| **Highest level of education** |  |  |  |  |
| *Postgraduate studies (master’s, doctorate, medical specialty, etc.)* | **Ref** |  |  |  |
| *Incomplete primary schooling* | **-** | - | - | - |
| Primary school | 1.31 | 0.741 | 0.27 | 6.47 |
| *Secondary school* | 0.62 | 0.472 | 0.17 | 2.28 |
| *Technical-professional training* | 1.05 | 0.929 | 0.38 | 2.90 |
| *Undergraduate degree (university training)* | 1.56 | 0.238 | 0.74 | 3.24 |
| **Living with someone < 18** |  |  |  |  |
| *No* | **Ref** |  |  |  |
| *Yes* | 0.67 | 0.236 | 0.34 | 1.30 |
| **Living with someone >65** |  |  |  |  |
| *No* | **Ref** |  |  |  |
| *Yes* | 0.73 | 0.415 | 0.33 | 1.57 |
| **Living with someone with disabilities** |  |  |  |  |
| *No* | **Ref** |  |  |  |
| *Yes* | 1.95 | 0.319 | 0.52 | 7.29 |
| **Profession** |  |  |  |  |
| *Non-clinical* | **Ref** |  |  |  |
| *Clinical* | 1.99 | 0.108 | 0.86 | 4.65 |
| **Beirut blast exposure^** | 1.001 | 0.148 | 0.99 | 1.00 |
| **Major accident (Vehicle, work-related, severe fall etc?)** |  |  |  |  |
| *No* | **Ref** |  |  |  |
| *Yes* | 2.55 | 0.046* | 1.02 | 6.40 |
| **Life-threatening personal physical illness** |  |  |  |  |
| *No* | **Ref** |  |  |  |
| *Yes* | 2.88 | 0.056 | 0.97 | 8.49 |
| **Life-threatening physical illness of a loved one/other** |  |  |  |  |
| *No* | **Ref** |  |  |  |
| *Yes* | 2.54 | 0.011* | 1.24 | 5.22 |
| **Death of loved one(s) not due to Beirut Blast** |  |  |  |  |
| *No* | **Ref** |  |  |  |
| *Yes* | 2.06 | 0.025* | 1.09 | 3.87 |
| **Ever exposed to war/conflict in Lebanon** |  |  |  |  |
| *No* | **Ref** |  |  |  |
| *Yes* | 0.77 | 0.419 | 0.40 | 1.46 |
| **Childhood physical abuse** |  |  |  |  |
| *No* | **Ref** |  |  |  |
| *Yes* | 3.19 | 0.001* | 1.61 | 6.33 |
| **Childhood neglect** |  |  |  |  |
| *No* | **Ref** |  |  |  |
| *Yes* | 3.16 | 0.002* | 1.55 | 6.46 |
| **Childhood sexual abuse** |  |  |  |  |
| *No* | **Ref** |  |  |  |
| *Yes* | 4.61 | 0.003* | 1.71 | 12.41 |
| **Financial Strain** |  |  |  |  |
| *No change* | **Ref** |  |  |  |
| *Yes change* | 0.79 | 0.490 | 0.41 | 1.53 |
| **Basic lifestyle** |  |  |  |  |
| *No change* | **Ref** |  |  |  |
| *Yes change* | 1.20 | 0.605 | 0.60 | 2.43 |
| **Leisure lifestyle** |  |  |  |  |
| *No change* | **Ref** |  |  |  |
| *Yes change* | 0.93 | 0.862 | 0.43 | 2.04 |
| **Number of household contributors** |  |  |  |  |
| *No change* | **Ref** |  |  |  |
| *Yes change* | 1.32 | 0.388 | 0.70 | 2.50 |
| **PTSD symptoms secondary to childhood trauma** |  |  |  |  |
| *No* | **Ref** |  |  |  |
| *Yes* | 38.75 | <0.001* | 8.06 | 186.36 |
| **PTSD symptoms secondary to wars, armed conflicts and explosions** |  |  |  |  |
| *No* | **Ref** |  |  |  |
| *Yes* | 1.61 | 0.245 | 0.72 | 3.58 |
| **PTSD symptoms secondary to a major accident** |  |  |  |  |
| *No* | **Ref** |  |  |  |
| *Yes* | 4.79 | 0.091 | 0.78 | 29.49 |
| **PTSD symptoms secondary to a life-threatening personal physical illness?** |  |  |  |  |
| *No* | **Ref** |  |  |  |
| *Yes* |  |  |  |  |
| **PTSD symptoms secondary to a loved one’s life-threatening physical illness** |  |  |  |  |
| *No* | **Ref** |  |  |  |
| *Yes* | 7.14 | <0.001* | 2.88 | 17.73 |
| **PTSD symptoms secondary to the death of a loved one (excluding the Beirut Port Blast)** |  |  |  |  |
| *No* | **Ref** |  |  |  |
| *Yes* | 2.35 | 0.042* | 1.03 | 5.34 |
| **Job reassignment/new functions since the pandemic** |  |  |  |  |
| *No* | **Ref** |  |  |  |
| *Yes* | 3.10 | 0.001* | 1.59 | 6.06 |
| **Covid-19 Score** | 1.04 | 0.072 | 1.00 | 1.09 |
| **Supportive colleagues’ network at work** |  |  |  |  |
| *Strongly disagree* | **Ref** |  |  |  |
| *Disagree* | 2.74 | 0.145 | 0.71 | 10.66 |
| *Agree* | 0.69 | 0.583 | 0.19 | 2.55 |
| *Strongly agree* | 0.35 | 0.191 | 0.07 | 1.70 |
| **Supportive loved one’s network** |  |  |  |  |
| *Strongly disagree* | **Ref** |  |  |  |
| *Disagree* | 2.00 | 0.577 | 0.18 | 22.80 |
| *Agree* | 1.33 | 0.790 | 0.16 | 11.09 |
| *Strongly agree* | 0.86 | 0.892 | 0.10 | 7.40 |
| **Need for psychological support due to the pandemic (even if not received)** |  |  |  |  |
| *No* | **Ref** |  |  |  |
| *Yes* | 2.44 | 0.007* | 1.27 | 4.65 |
| **Need for psychological support due to the financial situation (even if not received)** |  |  |  |  |
| *No* | **Ref** |  |  |  |
| *Yes* | 0.92 | 0.825 | 0.43 | 1.94 |
| **Need for psychological support due to the Beirut explosions (even if not received)** |  |  |  |  |
| *No* | **Ref** |  |  |  |
| *Yes* | 2.70 | 0.002* | 1.43 | 5.10 |
| **PTSD** |  |  |  |  |
| *No* | **Ref** |  |  |  |
| *Yes* | 0.71 | 0.438 | 0.30 | 1.67 |
| **Depression** |  |  |  |  |
| *No* | **Ref** |  |  |  |
| *Yes* | 5.07 | <0.001* | 2.44 | 10.54 |
| **Pre-pandemic chronic physical illness** |  |  |  |  |
| *No* | **Ref** |  |  |  |
| *Yes* | 1.25 | 0.624 | 0.51 | 3.11 |
| **Pre-pandemic mental health diagnosis** |  |  |  |  |
| *No* | **Ref** |  |  |  |
| *Yes* | 4.21 | 0.002* | 1.69 | 10.46 |
| **Change in use of tobacco (cigarettes, chewing tobacco, cigars, etc.) since the pandemic** |  |  |  |  |
| *I do not use this substance* | **Ref** |  |  |  |
| *My use has decreased* | 2.99 | 0.025* | 1.15 | 7.80 |
| *My use has remained the same* | 0.17 | 0.091 | 0.02 | 1.32 |
| *My use has increased* | 4.87 | <0.001* | 2.27 | 10.43 |
| **Change in use of alcoholic beverages (beer, wine, liquor, etc.) since the pandemic** |  |  |  |  |
| *I do not use this substance* | **Ref** |  |  |  |
| *My use has decreased* | 1.00 | 1.000 | 0.35 | 2.86 |
| *My use has remained the same* | 1.38 | 0.397 | 0.65 | 2.94 |
| *My use has increased* | 3.52 | 0.007* | 1.40 | 8.83 |

*^OR = odds ratio
CI = confidence interval^*

*^Ref = reference category^*

*^*p ≤ 0.05^*

*^^continuous variable^*

**Table S4: Simple logistic regression of lifetime suicide with COVID-19 variables**

| **Variable** | **OR** | **p-value** | **95% CI Lower** | **95% CI Upper** |
| --- | --- | --- | --- | --- |
| **COVID-19 exposure to suspected or confirmed cases (past week)** |  |  |  |  |
| *No* | **Ref** |  |  |  |
| *Yes* | 2.11 | 0.029* | 1.08 | 4.12 |
| **Patient deaths among COVID-19 cases under your care since the pandemic** |  |  |  |  |
| *No* | **Ref** |  |  |  |
| *Yes* | 2.56 | 0.019* | 1.17 | 5.60 |
| **Feeling stigma or discrimination as a health worker due to COVID-19** |  |  |  |  |
| *No* | **Ref** |  |  |  |
| *Yes* | 2.35 | 0.008* | 1.25 | 4.43 |
| **Extent of trust in workplace's ability to manage COVID-19^** | 0.27 | 0.003* | 0.12 | 0.65 |

*^OR = odds ratio
CI = confidence interval^*

*^Ref = reference category^*

*^*p ≤ 0.05^*

*^^continuous variable^*
